# Draft Genome Sequence of *Fervidicella metallireducens* Strain AeB^T^, an Iron-Reducing Thermoanaerobe from the Great Artesian Basin

**DOI:** 10.1128/genomeA.00345-14

**Published:** 2014-05-01

**Authors:** Bharat K. C. Patel

**Affiliations:** Microbial Gene Research and Resources Facility, School of Biomolecular and Physical Sciences, Griffith University, Brisbane, Queensland, Australia

## Abstract

The genome sequence of *Fervidicella metallireducens* strain AeB^T^, a curved, heterotrophic, thermoanaerobic, and iron-reducing bacterium isolated from a gray microbial mat colonizing the free-flowing waters of a Great Artesian Basin (GAB) bore well located in outback Queensland, Australia, is reported here. The analysis of the 2.9-Mb sequence indicates that the attributes of the genome are consistent with its physiological and phenotypic traits.

## GENOME ANNOUNCEMENT

*Fervidicella metallireducens* strain AeB^T^ (= KCTC 5667^T^ = JCM 15555^T^) was isolated from a gray microbial mat colonizing the free-flowing waters of a bore well in the Great Artesian Basin of Australia (1). The strain was cultured in a Trypticase yeast extract glucose medium under optimal conditions (pH 7.0 and 70°C). The cells were centrifuged, and the DNA from the cell pellet was purified using a modification of the Marmur method (2). The genomic DNA of *F. metallireducens* strain AeB^T^, sequenced using an Ion Torrent PGM sequencer and a 318 Chip at the Australian Genome Research Facility (AGRF) core facility, generated 1,212,514 reads totaling 235 Mbp. Genome assembly using the GS *de novo* Assembler (version 2.9) generated a total of 164 contigs (70× coverage). The assembled data of 2.9 Mbp, with an average G+C content of 32.04 mol%, were analyzed using the online annotation server RAST (3), and automatic gene annotation was carried out by the NCBI Prokaryotic Genomes Automatic Annotation Pipeline (PGAAP) (http://www.ncbi.nlm.nih.gov/genomes/static/Pipeline.html). The genome sequence comprises 2,800 putative protein-coding genes, 82 tRNA genes, and 6 rRNA genes (5S rRNA, 16S rRNA, and 23S rRNA). A limited number of genes involved in carbohydrate utilization (127) and a slightly higher number of amino acid degradation and synthesis genes (164) were identified in the genome. Genes involved in the uptake and transport of ferric ions, as well as genes for resistance to toxic ions, such as arsenic, cobalt, zinc, cadmium, copper, and zinc, were identified. Genes for protection against oxidative stress in anaerobic bacteria and genes involved in flagellar synthesis are also present. Analysis with the core gene data set of *F. metallireducens* strain AeB^T^ using PhyloSift version 1.0.0_02 (4) indicated that 71% of the phylogenetic marker genes are related to members of phylum *Firmicutes*. Other represented phyla include *Proteobacteria* (15%), *Actinobacteria* (4%), *Dictyoglomi* (2%), and *Spirochaetes* (2%). The closest phylogenetic relatives of the phylum *Firmicutes* were members of family 1, *Clostridiaceae*, with 54% of the markers related to *Caloramator australicus* strain R3^T^, 30% to members of the genus *Clostridium*, and the remaining 16% to other members of the family *Clostridiaceae*. The draft whole-genome sequence of *F. metallireducens* strain AeB^T^ will assist in understanding the physiology and adaptation of microbial life in the Great Artesian Basin of Australia, a deep geothermal subsurface multiaquifer.

### Nucleotide sequence accession numbers.

The whole-genome shotgun project of *F. metallireducens* strain AeB^T^ has been deposited at DDBJ/EMBL/GenBank under the accession no. AZQP00000000. The version described in this paper is version AZQP01000000.

## References

[1] Ogg CD Patel BKC 2010 *Fervidicella metallireducens* gen. nov., sp. nov., a thermophilic anaerobic bacterium from geothermal waters. Int. J. Syst. Evol. Microbiol. 60:1394–1400. 10.1099/ijs.0.014670-0 19671710

[2] Ogg CD Patel BKC 2009 *Caloramator australicus* sp. nov., a thermophilic, anaerobic bacterium from the Great Artesian Basin of Australia. Int. J. Syst. Evol. Microbiol. 59:95–101. 10.1099/ijs.0.000802-0 19126731

[3] Aziz RK Bartels D Best AA DeJongh M Disz T Edwards RA Formsma K Gerdes S Glass EM Kubal M Meyer F Olsen GJ Olson R Osterman AL Overbeek RA McNeil LK Paarmann D Paczian T Parrello B Pusch GD Reich C Stevens R Vassieva O Vonstein V Wilke A Zagnitko O 2008 The RAST server: Rapid Annotations using Subsystems Technology. BMC Bioinformatics 9:75. 10.1186/1471-2164-9-75 18261238 PMC2265698

[4] Darling AE Jospin G Lowe E Matsen FA Bik HM Eisen JA 2014 PhyloSift: phylogenetic analysis of genomes and metagenomes. PeerJ 2:e243–e243. 10.7717/peerj.243 24482762 PMC3897386

